# Reliability and Validity of a Lithuanian Version of the Oral Health Impact Profile—A Study in Patients with Stage III–IV Periodontitis

**DOI:** 10.3390/medicina59010069

**Published:** 2022-12-28

**Authors:** Eglė Zasčiurinskienė, Antanas Šidlauskas, Aistė Kavaliauskienė, Jurgita Vazgytė, Agnius Matuzas, Apolinaras Zaborskis

**Affiliations:** 1 Department of Orthodontics, Faculty of Odontology, Medical Academy, Lithuanian University of Health Sciences, LT-44307 Kaunas, Lithuania; 2 Department of Dental and Oral Pathology, Faculty of Odontology, Medical Academy, Lithuanian University of Health Sciences, LT-44307 Kaunas, Lithuania; 3 Faculty of Odontology, Medical Academy, Lithuanian University of Health Sciences, LT-44307 Kaunas, Lithuania; 4 Faculty of Public Health, Medical Academy, Lithuanian University of Health Sciences, LT-44307 Kaunas, Lithuania

**Keywords:** periodontitis, quality of life, oral health impact profile, translation, reliability, validity

## Abstract

*Background and Objectives*: The study aimed to translate the original English version of Oral Health Impact Profile (OHIP) into Lithuanian and to assess reliability and validity of the translated instrument (OHIP-Lt) in patients with advanced stages of periodontitis. *Materials and Methods*: Subjects (N = 67) with stage III–IV periodontitis aged 30–63 years were surveyed by questionnaire and examined clinically. Psychometric analysis included explanatory (EFA) and confirmatory (CFA) factor analyses and psychometric tests. *Results*: Cronbach’s alpha of the translated OHIP was 0.96. EFA revealed four dimensions which Cronbach’s alpha ranged from 0.75 to 0.96. Construct validity of the four-factor model derived from the OHIP-Lt was supported by findings of CFA (RMSEA = 0.077). The total OHIP-Lt and its subscale scores increased as the patients’ self-rated oral health status changed from healthy to unhealthy. Discriminative validity of the OHIP-Lt was confirmed by its higher scores among patients who had an increased spacing between the maxillary anterior teeth and increased clinical attachment level (CAL ≥ 5 mm) compared to those who did not. *Conclusions*: The translated Lithuanian version of OHIP-Lt was identified as four-dimension inventory. Good reliability and validity of the OHIP-Lt provide the evidence for its further use in study on advanced periodontal disease burden among Lithuanian patients.

## 1. Introduction

Oral health is an important component of a person’s quality of life [1,2,3]. Any pathophysiologic changes in the orofacial system lead to impairment and limitation not only in masticatory function but also in psychosocial well-being. Psychosocial impact, together with the dimensions of oral function, orofacial pain and dental appearance, has been proposed to cover the different areas of oral health-related quality of life (OHRQoL), the concept of which is widely acknowledged [4].

The Oral Health Impact Profile (OHIP) is the most commonly used questionnaire to measure the patient-perceived impact consistently across different oral health conditions [5]. The OHIP was originally developed and evaluated by Slade and Spencer in 1994 using the original Australian data set of persons [5]. Up to date, the OHIP has been translated into multiple languages, e.g., German [6], Spanish [7], Swedish [8], Chinese [9], Hungarian [10], Japanese [11,12], and evidence for the instrument’s cross-cultural equivalence is available [13,14]. It was further developed into several versions with a smaller number of items as compared to the original 49-item of the OHIP, such as the 14-item version [15]. Generally, seven dimensions of OHRQoL are incorporated in OHIP, covering functional limitation, physical pain, psychological discomfort, physical disability, psychological disability, social disability, and handicap, but despite that dimensionality of OHIP has been discussed [13,14]. The OHIP was validated in different target populations, e.g., in prosthodontic patients [10,13,14], malocclusion patients [8], patients with removable dentures [11], edentulous subjects [12], among the elderly [9]. In the literature, special attention is paid to the measurement of OHRQoL in patients with periodontal disease and to the application of the OHIP inventory for this purpose [16,17,18,19,20].

Periodontitis is one of the most common dental diseases with a high prevalence (up to 50%), were its severe forms affect 11.2% of the world’s population [21]. Research in Lithuania also shows its high prevalence: a quarter (24.3%) of middle-aged and elderly subjects required complex treatment for periodontal disease [22]. It is well described in the literature that advanced stages of the disease lead to the destruction of the periodontal tissues which cause tooth mobility, sensitivity, and, if not treated, loss of multiple teeth. Therefore, daily living of these subjects is affected by variety aesthetic, functional, physical, and psychosocial limitations, which have impact on deterioration of their OHRQoL.

Although scientific literature on the relationship between the severity of periodontitis and patients’ quality of life has a long history, the negative impact of periodontal disease on OHRQoL has been investigated less than other oral problems, such as dental caries and tooth loss [18]. There is still low progress in the development of condition-specific instruments of OHRQoL measure to be used in subjects with advanced stages of periodontitis, observed by assessing the significance of individual items or the dimensionality of the whole instrument [17]. In Lithuania, so far only 14-item OHIP form has been tested for its reliability and validity [23]. Due to the lack of the literature and at the same time the increasing interest in the multidisciplinary treatment of subjects with advanced stages of periodontitis our vision was to apply 49-item OHIP questionnaire to aforementioned subjects to find out possible new predictors of these advanced (stage III–IV) stages of the disease.

This study aimed to translate the original English version of OHIP into Lithuanian and to assess psychometric properties of the translated instrument (OHIP-LT) to substantiate its validity in measuring OHRQoL in patients with stage III–IV periodontitis.

## 2. Materials and Methods

### 2.1. Ethics and Consent to Participate

The study was conformed to the principles outlined in the Declaration of Helsinki. Ethical approval for the study was granted by the Kaunas Regional Biomedical Research Ethics Committee (protocol code P1-BE-2-111-2019 approved on 15 March 2021). Additionally, written informed consent was obtained from all subjects involved in the study.

### 2.2. Study Sample

The study followed a cross-sectional design. The minimum sample size was estimated from the position of conducting factor analysis. The literature states that the sample size in factor analysis is related to the number of variables and factors, and the size of the communalities [24]. The OHIP questionnaire contains 46 items with 4 expected factors (variable-to-factor ratio = 11.5). Under these conditions and assuming that all communalities have a wide range (from 0.2 to 0.8), in order to conduct a reliable factor analysis, according to the recommendations by Mundfrom et al. [24], the minimum sample size should be 60.

The study targeted adults aged 30 and older. Patients who referred to Orthodontic department at Lithuanian University of Health Sciences (March 2021–January 2022) were invited to participate in the study asking them to fill the OHIP questionnaire and undergo periodontal-orthodontic examination. Subjects were introduced to the purposes, tasks and the course of biomedical research. The subject had the right to refuse to participate in the investigation or to terminate his participation at any time without explanation. Inclusion criteria involved: diagnosed with stage III or IV periodontitis, 30 years of age and older. Exclusion criteria were: stage I–II periodontitis, non-inflammatory periodontal disease, removable prosthetic appliances, multiple missing anterior teeth, pregnant/lactating women, smoking >20 cigarettes/day, and oncologic or diabetes diagnosis in patient’s history.

Of 121 invited patients who suffered of stage III–IV periodontitis and met other all inclusion and exclusion criteria, 74 underwent intraoral examination and questionnaire survey, so the response rate was 61.2%.

### 2.3. Measures

#### 2.3.1. OHIP Questionnaire

*Translation and pre-testing*. The original OHIP questionnaire developed by Slade and Spencer (1994) [5] includes 49 items. In the present study, due to exclusion criteria, three questions, asking about denture-related problems, were omitted. Therefore, the English version of the 46-item OHIP was used as described in the study by Adulyanon and Sheiham [25] and compared with the versions earlier used in other validation studies [13,14].

The procedure of translation and national adaptation of the questionnaire followed guidelines proposed by Beaton et al. [26] and the principles of good practice outlined by International Society for Pharmacoeconomics and Outcomes Research [27]. It was first forward translated into Lithuanian by a co-author (EZ) of this study, who was familiar with the concepts included in the OHIP. Her mother-tongue language is Lithuanian and she is fluent in English. During this phase, the main focus was to achieve semantic, idiomatic, conceptual and scientific equivalence between the English and Lithuanian versions while adopting a vocabulary easily comprehensible. Then, the translated questionnaire was reviewed by all study co-authors. Later, the Lithuanian version of the OHIP was blindly back-translated into the English language by a professional translator, fluent in English and unfamiliar with the concepts of the OHRQoL and original English version. The back-translated English questionnaire was compared to the original one, aiming to discern possible discrepancies and to solve any inconsistencies between the two versions. All study co-authors, and the back-translator discussed the differences between the initial English and Lithuanian versions of the questionnaire. A consolidated Lithuanian version (OHIP-LT) was approved by consensus.

Prior to the main study, a pilot test was carried out on a sample of adult orthodontic patients (*n* = 12) in the university clinic. It was aimed to verify the level of understanding of the wording used and, where appropriate, to make any necessary changes. This so called “face validity” test confirmed the feasibility of the methodology and showed that the patients can understand the questions.

*Coding and scoring*. To facilitate comparison of results, we used the same question numbers and their sequence in the OHIP questionnaire as they were presented in studies by John et al. [13,14]. Respondents were asked to indicate on a five-point Likert scale how frequently they experienced each problem within the recent 12 months period. Response categories for the five-point scale were: “very often”, “fairly often”, “occasionally”, “hardly ever” and “never”. Respondents may also be offered a “don’t know” option for each question. In analyzes, responses were ranked and coded as follows: 0—”never”, 1—”hardly ever”, 2—”occasionally”, 3—”fairly often”, and 4—”very often”. “Don’t know” responses were recoded to missing values. If more than 9 responses (approximately 20% of all questions) had missing values, than the questionnaire was removed from the analysis. Due to this reason 7 questionnaires were removed, thus in the remaining 67 out of 74 subjects, missing responses were imputed with the mean value of all valid responses to the corresponding question.

OHIP-LT scores have been computed in three methods. First, the simplest method was to count the number of responses “fairly often” or “very often” for each subject. The second estimate was sum score that summed up all scores of the answers for each subject. Using the third method, scores of the answers were multiplied by the corresponding weight for each question and the products were summed. In other words, the factor weights of the whole scale or its individual dimensions were calculated using this method. Note that higher estimates of OHIP scores refer to worse OHRQoL.

*Self-rated dental health and demographic variables.* During the questionnaire survey, the patients were also asked to rate their dental health: “In overall, how do you rate your dental health?”. Response options were: 1—”excellent”, 2—”fairly good”, 3—”average”, 4—”fairly poor”, and 5—”very poor”. The question was intended to be used in the convergent validity analysis.

Patients were grouped by sex (male, female) and age. Age was assessed as a continuous variable. As prevalence of severe forms of periodontitis has been described to reach its peak at age 40 years, for this study, we transformed subjects’ age into two categories of age groups (≤40 and >40 years) [20].

#### 2.3.2. Oral Examination and Measures

All subjects who filled the questionnaire participated in a clinical oral examination. Clinical attachment level (CAL) was chosen as the most important periodontal variable, which was measured as a distance from the bottom of the pocket to cement-enamel junction and recorded in millimetres [28,29]. CAL was recorded at six points per tooth for all teeth using a manual periodontal probe (Hu-Friedy PCP-UNC 15, Chicago, IL, USA). Measurement analysis included sites that had CAL ≥ 5 mm describing the severity periodontitis [30]. In addition, the percentage of sites CAL ≥ 5 mm within each subject was calculated describing the extent of the disease. Patients were divided into two groups using 30% cut-off point. Stage (III or IV) and grade (A, B or C) of advanced periodontitis was assessed by two experienced periodontists as described in the new classification and case definition [31]. Any disagreements were solved by discussion.

The second measure assessed the pathologic migration of anterior teeth due to the aggressive periodontal process [29,30,32]. Pathologic teeth migration was defined as a change in the position of a tooth that occurred when the balance of forces, which maintains it in its normal position, was broken. All types of pathological dental migration, including diastema (spacing ≥ 1 mm), were recorded.

All measurements were performed by only one examiner (EZ). Calibration of the examiner for periodontal measurements was performed in the previous study [33].

### 2.4. Statistical Analysis

#### 2.4.1. Descriptive Statistics

The distributions of each item and the sum scores were examined. The sum scores and other estimates of OHIP were found not to be normally distributed, thus, the equity of their distributions between groups was tested using non-parametric tests. Upon the same reason, binary associations between variables were evaluated with non-parametric Spearman correlation coefficient.

#### 2.4.2. Psychometric Properties

A set of tests was used for examining psychometric properties of the inventory [34,35,36]. The Cronbach’s alpha and intraclass correlation coefficient (ICC) average measure (one-way random effects) [37] were used as a measure of internal consistency reliability of the total instrument and its domains. Values less than 0.5, between 0.5 and 0.75, between 0.75 and 0.9, and greater than 0.90 were indicative of poor, moderate, good, and excellent reliability, respectively [36]. Furthermore, other tests of internal reliability (item-total correlations and Cronbach’s alpha if item deleted) were also investigated. Convergent validity of the instrument was tested using Spearman correlation coefficient to assess the association between the scores of total scale, as well as its domains and the respondents’ rating of their dental health (excellent, fairly good, average, fairly poor, very poor). Discriminative validity was tested by comparing the distributions of scores between groups defined by sex, age, and clinical dental health traits.

#### 2.4.3. Factorial Validity

*Exploratory factor analysis* (EFA) identifies the structure/dimensionality of observed data to reveal the underlying constructs of observed phenomena; it results in the smallest and most compatible number of underlying factors from a larger set of initial variables on a questionnaire [36,38].

Using the SPSS Principal Component Factor Analysis procedure, an EFA was performed on the set of OHIP items. The suitability of the data for such analysis was tested using the Kaiser-Meyer-Olkin (KMO) measure of sampling adequacy along with the Bartlett’s test of sphericity (KMO ≥ 0.5 and *p* < 0.001 show the adequacy of the data for use in the EFA). Initially, we explored single-factor solution that ranks the items by their impact to the total variance of the scale. Then, the multidimensionality of the scale was judged from the ratio of the first and second eigenvalue. If this ratio was less than 4, it could be assumed that the data set is better described by more than one factor [39]. To determine how many factors to be extracted we based on the eigenvalue (>1) criteria and a Cattell’s scree plot. A *Promax oblique* rotation was employed. This procedure led to the calculation of the factor loadings for each question item in a meaningful way, since factors were correlated [35]. Factor loadings less than 0.4 indicated low item impact on the validity of the instrument [36,38], therefore, further analysis was performed without such items.

*Confirmatory factor analysis* (CFA) [36,40,41,42] was employed to establish factorial validity of the OHIP dimensions. Initial model was based on the above EFA postulating that the model should have as many latent variables as many factors were established and that latent variables might be correlated. The goodness of fit of the explored models was evaluated using multiple fit indices. Relative chi-square (*χ*^2^/df) and its *p*-value, Comparative Fit Index (CFI), Tucker–Lewis Index (TLI) and Root Mean Square Error of Approximation (RMSEA) were taken into account. Relative chi-square is the chi-square ratio to degrees of freedom, and it is suggested that its value less than 3 or a non-significant *p*-value corresponds to an acceptable fit, however, the chi-square increases with sample size and model complexity and, therefore, this test was complemented by other tests [42]. The values of CFI and TLI values close to 1 (≥0.90) are commonly indicated as acceptable model fits [36,42]. A RMSEA value between 0.08 and 0.10 indicates an average fit, and a value below 0.08 and below 0.05 shows correspondingly a good and excellent fit [36,42].

All analyses were conducted using the SPSS statistical package supplemented with AMOS (version 21; IBM SPSS Inc., Chicago, IL, USA, 2012). The cut-off level for statistical significance was set at 0.05.

## 3. Results

### 3.1. Sample Characteristics

A total, 74 subjects filled the OHIP questionnaire. However, seven questionnaires had ≥20% missing values and therefore were removed from the final analysis. Therefore 67 subjects (31.6% men and 68.4% women) were included and clinically examined. Their age range was 30–63 years (mean 43.9, median 42, SD 8.8); 45.6% were ≤40 years of age and 54.4% were >40 years of age. Of them, 67.2% subjects were diagnosed with stage III (40.0% grade A, 28.9% grade B and 31.1% grade C) and 32.8 % with stage IV periodontitis (9.1% grade A, 9.1% grade B and 81.8% grade C).

The majority of patients rated their dental health as fairly poor (26.9%) or very poor (37.3%). An objective clinical examination identified CAL ≥ 5 mm in more than 30% of dental surfaces among 22% of patients. A noticeable dental spacing between anterior maxillary teeth was found among 58% of patients.

### 3.2. Analysis of OHIP Items

Table 1 demonstrates psychometric characteristics of each question from the OHIP-LT questionnaire. A high variety was observed in evaluating the mean scores of the answers to the questions and, consequently, the skewness of the answer scores distribution. For some questions (e.g., V2, V25, V26, V39, V40, V48), the majority of respondents chose the answers “never”, while in answering some other questions (e.g., V3, V7), many respondents tended to report that the problems identified in the questions occur to them “very often” or “fairly often”. The first group of items had low mean values and very positive skewness, the second group of items had great mean values and very negative skewness, but in both cases the items had the lowest standard deviations. Correlation between item score and total sum score ranged from 0.28 (questions V5 and V7) to 0.76 (questions V35 and V47). Cronbach’s alpha of the total OHIP instrument was 0.96. This value changed little if any item was deleted from the scale.

Summarizing the findings of this analysis, it can be concluded that OHIP-LT includes some specific questions on OHRQoL for subjects with stage III–IV periodontitis. Some questions were of little significance in the scale if they were characterized by the following psychometric properties: a very rare occurrence of complain (low mean value, low proportion of response options “fairly often” and “very often”) or a very high occurrence of complain (high mean value, high proportion of answers “very often” and “fairly often”), low standard deviation, high positive or negative skewness, low item-total correlation, small impact on Cronbach’s alpha. In addition, these items had low loading in the single-factor solution (see next subsection). In subjects with stage III–IV periodontitis, many of these features were specific to the following questions: V2 Trouble pronouncing words, V3 Noticed tooth which doesn’t look right, V5 Breath stale, V7 Food catching, V48 Unable to function.

### 3.3. Exploratory Factor Analysis

Construct validity of OHIP was investigated by exploratory factor analysis (EFA). The adequacy of the data for such analysis was confirmed by a KMO test with a value of 0.71 above the recommended threshold value of 0.50, and by significance (*p* < 0.001) of the Bartlett’s test of sphericity.

Table 2 presents results of exploratory factor analysis of the OHIP-LT. The factor loadings of 46 items on a single-factor ranged from 0.29 (item V5 Breath stale) to 0.79 (item V47 Life unsatisfying), of them 35 loadings were above 0.50. A single factor explained 34.7% of the total variance, however, the analysis of the dimensionality of the scale revealed 22 components with eigenvalue >1, making up 91.9% of the total variance. Based on Cattell’s scree plot, there were four eigenvalues above the elbow of the plot, consequently it was decided to retain four factors, which altogether accounting for 54.4% of the total variance. The ratio of the first and second eigenvalues was 3.19 (15.48/4.85 = 3.19). Although this ratio indicates the dominance of the first factor, but its values less than 4 suggests that a one-dimensional data description is not sufficient [39].

Then, the loadings of four factors for each item after *Promax* rotation were calculated (see Table 2). The first factor contained 14 items mainly addressed psychosocial issues; therefore, it was labelled “*Psychosocial Impact*”. The item V45 Financial loss has also been assigned to the first factor, but due to small loadings, this item was not significant for the factor. The second factor comprised 15 questions mainly related to functional limitations, hence it was called “*Functional Limitation*”. Among the items of the second factor stands out V3 Noticed tooth which does not look right, which had a negative loading, showing the opposite effect on functional limitations, but having a significant direct effect on the next factor. The third factor included 13 questions that reflect concerns about pain in the orofacial system, leading to the name “*Orofacial Pain*”. Finally, the fourth factor contained only four items, three of which directly reflected concerns about *Speech Limitations*. Several items (e.g., V3, V25, V32, V37, V44) had great loading values for alternative factors as well; therefore, they might have an impact to more than one specified factor. The four factors correlated between 0.33 and 0.48. Twelve items (1 item of factor 1, 6 items of factor 2, and 5 item of factor 3) did not have salient (≥0.50) loadings on any of the four factors. Their use in further analysis could be inexpedient, for instance, Cronbach’s alphas did not change noticeable if these items were excluded from the corresponding subscales (see Table 1).

### 3.4. Confirmatory Factor Analysis

The dimensionality of the OHIP-LT was further investigated employing confirmatory factor analysis (CFA). The CFA models assigned items to factors (dimensions) under the classification suggested by the EFA. The items with loadings <0.50 were excluded from the analysis, thus, 37 items were added to the single-factor model, and 34 items were added to the four-factor model. Table 3 reports standardized factor loadings and fit statistics for explored CFA models.

In single-factor CFA model, all standardized factor loadings were statistically significant (*p* < 0.05) ranging from 0.38 (V17 Sore spots) to 0.84 (V35 Difficult to relax). However, the single-factor model fit the data poorly, despite its improvement through modification indices. All fit statistics did not indicate acceptable model fit, suggesting that the OHIP-LT covariance structure cannot be well-modelled by a latent single-factor model.

In four-factor CFA model, the majority of standardized factor loadings increased over the single-factor model, and reached a value of 0.98 (V24 Speech unclear) in Speech *Limitations* factor. The estimated correlations inter-factors ranged between 0.36 and 0.68 similarly to the findings from EFA. Factors *Functional Limitations* and *Orofacial Pain* showed the strongest correlation. The four-factor model fit the data significantly better than the single-factor model, as evidenced by a likelihood ratio test: *χ*^2^(80) = 617, *p* < 0.001. Although the chi-square test rejected the four-factor model (*χ*^2^(480) = 670, *p* < 0.001), but the ratio *χ*^2^/df = 1.40 did not exceed the recommended limit 2. Other model fit indices also improved (CFI = 0.922; TLI = 0.909; RMSEA = 0.077 with 90% CI [0.063, 0.091]) over the single-factor model indicating acceptable model fit to data. Thus, based on the high factor loadings and significant improvement in model fit, it can be argued that OHIP data in patients with stage III–IV periodontitis have four dimensions.

### 3.5. Internal Consistency and Reliability Analysis

Cronbach’s alpha and intraclass correlation coefficient (ICC) were the main indicators for OHIP-LT reliability analysis. Cronbach’s alpha resulted in 0.96 for the total scale, indicating an excellent internal consistency (Table 4). Excellent or good internal consistency were also found for subscales of items determined by four-factor solution. These findings were supported by ICC which for the total OHIP-LT was 0.95. The ICCs of the four subscales ranged between 0.48 and 0.94. Approximately the same estimates of internal consistency were achieved in analysis of data sets without items which had no salient factor loadings.

### 3.6. Convergent Validity

To demonstrate evidence of convergent validity, we tested the hypothesis that OHIP-LT is related with other scales that measure a similar construct. In this study, self-reported dental health was considered as an alternative measure of OHRQoL. Table 5 presents Spearman’s correlations between the estimates of OHIP-LT and self-reported dental health scores.

The subjective estimation of dental health strongly correlated with all estimates of overall OHIP-LT (the value of the Spearman’s correlation coefficient varied between 0.70 and 0.74). By dimensions, it had the strongest correlation with the *Psychosocial Impacts* (*ρ* = 0.74) and substantial magnitude correlation with the *Orofacial Pain* (*ρ* = 0.51), while correlation with the *Speech Limitations* was weak (*ρ* = 0.23).

### 3.7. Discriminative Validity

Discriminative validity of the OHIP-LT instrument was tested by comparing the scale scores in groups of subjects by sex and age, as well as by clinical dental status (CAL and spacing). Three estimates of the scale scores (percentage of answers “fairly often” and “very often”, sum score, and general factor score) were employed to assess difference in responses to all OHIP-LT questions. In addition, it was determined whether all four dimensions depend on demographic and clinical variables. Results of discriminative validity analysis are presented in Table 6.

A significant sex difference was revealed only for the dimension of *Psychosocial Impact*; females were more likely to report higher frequencies of psychosocial complains. A significant gradient across the groups of patients by age (worse OHRQoL in younger than in older group) was observed by assessing the overall OHIP-LT and the dimension of *Psychosocial Impact*, meanwhile, the remaining three dimensions were independent of age. Patients with CAL ≥5 mm in more than 30% of dental surfaces comparing with their counterparts were more likely to report worse OHRQoL but in case of their functional limitation issues the difference between group of patients was insignificant. Patients with increased spacing between teeth rated their overall OHRQoL as well as dimensions of *Psychosocial Impact* significantly worse, but did not differ from other patients in terms of the remaining three dimensions. In overall, a discriminative validity analysis show that the dimension of *Psychosocial Impact* was related to all variables examined in this analysis.

## 4. Discussion

In this study, analysis of psychometric properties of the whole translated instrument and its four-factor dimensions provided evidence of Lithuanian OHIP validity in measuring OHRQoL in patients with advanced stages of periodontitis. Considering this point, our study is innovative in OHRQoL research in Lithuania and also worldwide, as literature on the topic is scarce [17].

As discussed in the method section, we used only 46 questions because we excluded 3 questions for subjects with removable dentures due to exclusion criteria. The contribution of each of the 46 OHIP questions to the overall OHRQoL was assessed. The significance of the scale items was not uniform; single-factor analysis has shown that questions about psychological well-being (e.g., V35 Difficult to relax, V47 Life unsatisfying) were the most important. We could compare the order of significance of items found in the present study with the results of the international study of general adult population and subjects with prosthodontic treatment need [13]. Single-factor loadings found in the present study seemed strongly correlated (r = 0.70) with the results of the aforementioned comparative study. It was, however, noticed that some OHIP items have exceptional psychometric characteristics. Items V3 Noticed tooth which doesn’t look right, V5 Breath stale, and V7 Food catching had low loadings both in our study and the comparative study. However, items V2 Trouble pronouncing words, and V48 Unable to function were different in values of single-factor loadings comparing our data with a total sample as well as with separate samples of six countries that were involved into John et al. cross-national study [13]. Nevertheless, it is difficult to find arguments that such a difference could be due to different oral pathologies.

The OHIP was originally composed of seven distinct constructs of oral health [5,6,8,11] but this structure has been consistently rejected in subsequent studies [43]. The study in the six countries by John et al. [13] revealed four dimensions and the strong general factor structure of the 46-item OHIP. In our study, EFA and CFA confirmed the four dimensions structure of OHIP-LT that was in line with John’s et al. study in respect of four-factor dimensionality of the instrument. In both studies, the dominant (first) factor was *Psychosocial Impacts*, but the content and order of the remaining three factors differed. The psychosocial component was singled out in all OHIP validation studies [11,17]. In addition, our data showed a defined *Speech Limitations* factor that has not been yet identified in other studies. In contrast to John’s et al. study [13], the one-factor design of our study data proved to be too poor, so it can be used as an aid in assessing the weight of individual items or the sum score on the full scale.

The next step in this analysis was to test the reliability and validity of the whole OHIP-LT and its dimensions. In the previous studies, a wide range of statistical tools have been used for validity and reliability analysis of translated OHIP containing 49 or 14 items. So it is difficult to compare the validity results between studies because of the methodological differences among investigations. Different populations make also comparisons difficult to interpret. However, there seems to be a consensus across the studies that the original instrument translated into other languages has been rated positively as a precise, valid and reliable instrument [6,7,8,9,10,11,12]. The findings of the present study also confirmed high internal consistency of the original OHIP with the national data. Cronbach alpha for the total OHIP was 0.96, which is in accordance with that reported by the developers of the instrument in their validation study [5]. In general, the internal consistency of the present survey was in any case as high as that reported by other OHIP validation studies [6,7,8,9,10,11,12]. The lowest Cronbach alpha (0.75) was found for the *Speech Limitations*. Since the value of Cronbach alpha depends on the number of items that make up the scale [35], its lowest value can be explained by the fact that this subscale contains only four items.

A discriminative validity is another important psychometric assessment of scales which shows the degree to which a measure diverges depending on another measures [36]. It appeared that OHIP-LT was able to discriminate between demographic groups by sex and age. Sex-related significant difference in patients was found only in the dimension of *Psychosocial Impacts*; women were more likely to report oral illness of a psychosocial nature than men. This finding is in accordance with conclusions reported by several other authors [23,44,45]. Difference in the perception of psychosocial impacts of OHRQoL on daily activities between the sexes may be caused by individual and subjective concepts related to beauty and aesthetic standards, imposed by the social demands and personal needs [45]. Age-related difference we found also in the dimension of *Psychosocial Impacts*, but due to its high value, the effect occurred across the total scale as well. It is natural for younger subjects to have better oral health, but it turns out that they are more likely than older people to experience oral health problems and underestimate their OHRQoL. However, this controversial phenomenon has not yet been sufficiently described in the literature. Meanwhile, Sierwald et al. [46] in the assessment of oral problems among prosthodontic patients claimed that OHIP was not related significantly neither with age nor sex.

For the discriminative validity, significant differences in the mean scores of the total OHIP-LT and its subscales were shown among patients with varying severity of chronic periodontitis. According to some studies [30,32,47,48] the periodontal pockets and pathological migration of teeth are the most common complaints that lead periodontal patients to seek treatment. The resent clinical studies of advanced stages of periodontal disease evaluated the potential outcomes of implant rehabilitation of diabetic patients [49], and of patients with bone atrophy to periodontal disease [50,51]. Moreover, pathologic tooth displacements may aesthetically as well as functionally and emotionally affect the subjects [32]. We found higher OHIP-LT scores in subjects with stage III–IV periodontitis where more than 30% of tooth surfaces were affected by CAL ≥ 5 mm. Pathologic tooth migration, such as spacing between the maxillary anterior teeth was another important dental trait that negatively affected OHRQoL, specifically its psychosocial dimension. Based on these results, it appears that Lithuanian version of OHIP is valid in distinguishing patients with stage III–IV periodontitis. This finding was consistent with other OHIP validation studies [7,17].

### Strengths and Limitations

In terms of strengths, the present study is the first OHIP validation study conducted in Lithuania, which includes cross-cultural adaptation procedures for the critical validation process [26]. We fulfilled guidelines translating the original questionnaire into Lithuanian, including back translation [27] and verified the level of understanding of the wording used by a ‘face validity’ test in a limited group of patients. Then, a group of patients with advanced stages of periodontitis were surveyed both with OHIP-LT instrument and intraoral examination. Exploratory and confirmatory factor analyses confirmed a four-factor structure of the translated instrument.

Still, limitations of the study also need to be considered. One of the limitations of the present study includes its relatively small size, presumably due to reluctance of the subjects to participate. It is worth to mention that the study was performed during COVID-19 pandemic and many of the relevant subjects were lost as they did not come to comprehensive clinical examination. Another factor is that we included only subjects with stage III–IV periodontitis which is only 11 per cent of the population [21]. However, the number of subjects available was sufficient to obtain a high level of significance, for example, in testing differentiate validity. Second, the generalizability of this study may be somewhat limited due to the restrictive nature of the sample, which included only subjects with advanced stages of periodontitis. Our sample differed from general population or samples of subjects with earlier stages of periodontal disease. Also, we did not include control group as this, due to COVID-19 pandemic, was practically impossible. Therefore, future research studies should test the reliability and validity of the Lithuanian OHIP in different populations. Third, we conducted EFA and CFA on the same sample of patients. In regard with this issue there is a hot debate between researchers [52]. A part of researchers argues that combining EFA and CFA allows a rigorous assessment of the instrument properties, i.e., EFA and CFA are not in conflict with each other, rather can be used complimentary to each other and can be used in the same data set. Such a solution is important in testing the questionnaire verified in previous studies, after all, by translating the questionnaire into another language. This is a case of the present study. The other part of researchers is following the alternative that EFA and then CFA must be performed on different samples drawn from the same population as this yields high danger of overfitting, in particular in smaller datasets [52]. Identification and verification of factors on the separate samples would be helpful to generalize the results. Fourth, a test-retest reliability test to evaluate the stability of the instrument and the assessment of the responsiveness of OHIP-LT to changes in oral health conditions were not employed because of limited number of appointments with the patients.

## 5. Conclusions

The translated Lithuanian version of OHIP was identified as four-dimension instrument. Good reliability and validity properties of the OHIP-LT provide the evidence for its further use in study on periodontitis-specific aspects of OHRQoL among Lithuanian patients.

## Figures and Tables

**Table 1:** Psychometric characteristics of the OHIP-LT items (*n* = 67).

OHIP-LT Item	Mean	Standard Deviation	Skewness	Percentage of Response Options “Fairly Often” and “Very Often”	Corrected Item-Total Correlation	Cronbach’s Alpha If Item Deleted
V1 Difficulty chewing	1.61	1.17	0.10	19.4	0.50	0.954
V2 Trouble pronouncing words	0.48	0.89	1.91	3.0	0.32	0.955
V3 Noticed tooth which doesn’t look right	3.25	0.91	−1.15	79.1	0.31	0.955
V4 Appearance affected	2.34	1.51	−0.45	50.7	0.61	0.954
V5 Breath stale	2.10	1.12	−0.21	31.3	0.28	0.955
V6 Taste worse	0.64	1.00	1.83	7.5	0.55	0.954
V7 Food catching	3.10	1.00	−1.24	79.1	0.28	0.955
V8 Digestion worse	1.03	1.23	1.10	16.4	0.60	0.953
V10 Painful aching	1.60	1.30	0.21	17.9	0.35	0.955
V11 Sore jaw	0.72	1.01	1.15	9.0	0.42	0.954
V12 Headaches	0.76	1.06	1.52	10.4	0.49	0.954
V13 Sensitive teeth	2.42	1.00	0.09	41.8	0.39	0.955
V14 Toothache	2.03	0.95	0.15	22.4	0.42	0.954
V15 Painful gums	1.96	1.12	−0.11	23.9	0.56	0.954
V16 Uncomfortable to eat	1.87	1.21	−0.11	28.4	0.63	0.953
V17 Sore spots	1.70	1.14	0.18	20.9	0.50	0.954
V19 Worried	2.82	1.19	−0.80	64.2	0.63	0.953
V20 Self–conscious	2.37	1.32	−0.44	46.3	0.65	0.953
V21 Miserable	2.40	1.29	−0.50	49.3	0.72	0.953
V22 Uncomfortable about appearance	2.36	1.41	−0.50	53.7	0.63	0.953
V23 Tense	2.09	1.30	−0.17	38.8	0.64	0.953
V24 Speech unclear	0.60	0.87	1.32	4.5	0.66	0.953
V25 Others misunderstood	0.39	0.82	2.10	4.5	0.60	0.954
V26 Less flavour in food	0.36	0.85	2.62	4.5	0.57	0.954
V27 Unable to brush teeth	1.99	1.21	−0.34	38.8	0.39	0.955
V28 Avoid eating	1.72	1.31	0.13	23.9	0.57	0.954
V29 Diet unsatisfactory	0.96	1.25	1.20	16.4	0.51	0.954
V31 Avoid smiling	2.07	1.45	−0.17	38.8	0.63	0.953
V32 Interrupt meals	0.69	1.02	1.38	7.5	0.68	0.953
V33 Sleep interrupted	0.60	0.94	1.69	6.0	0.52	0.954
V34 Upset	2.04	1.30	−0.09	35.8	0.65	0.953
V35 Difficult to relax	1.63	1.29	0.17	23.9	0.76	0.953
V36 Depressed	1.70	1.31	0.12	26.9	0.66	0.953
V37 Concentration affected	0.99	1.15	0.90	11.9	0.69	0.953
V38 Been embarrassed	1.87	1.33	0.01	31.3	0.66	0.953
V39 Avoiding going out	0.27	0.62	2.55	1.5	0.57	0.954
V40 Less tolerant of others	0.27	0.69	2.77	3.0	0.46	0.954
V41 Trouble getting on with others	0.94	1.18	1.03	10.4	0.60	0.953
V42 Irritable with others	0.73	0.95	1.01	6.0	0.63	0.953
V43 Difficulty doing job	0.61	0.90	1.24	4.5	0.63	0.953
V44 Health worsened	0.76	0.99	1.29	7.5	0.70	0.953
V45 Financial loss	1.24	1.38	0.80	20.9	0.48	0.954
V46 Unable to enjoy people’s company	0.96	1.13	0.99	7.5	0.60	0.954
V47 Life unsatisfying	1.07	1.17	0.84	11.9	0.76	0.953
V48 Unable to function	0.19	0.53	3.35	1.5	0.38	0.955
V49 Unable to work	0.54	0.86	1.36	3.0	0.49	0.9574

Note: OHIP-LT: Oral Health Impact Profile, Lithuanian version.

**Table 2:** Results of exploratory factor analysis: factor loadings in the single-factor solution and four-factor solution for 46 items of the OHIP-LT.

Factor Name and Item	Standardized Factor Loadings				
	Single-Factor Solution	Four-Factor Solution			
		Factor 1	Factor 2	Factor 3	Factor 4
Factor 1. *Psychosocial Impact*					
V34 Upset	0.67	0.93	0.07	−0.04	−0.23
V22 Uncomfortable about appearance	0.65	0.92	−0.23	−0.03	0.15
V20 Self-conscious	0.66	0.91	−0.13	−0.06	0.08
V21 Miserable	0.74	0.88	−0.10	0.03	0.10
V36 Depressed	0.69	0.87	0.22	−0.13	−0.18
V38 Been embarrassed	0.67	0.86	−0.07	0.02	0.00
V23 Tense	0.66	0.84	0.06	−0.17	0.08
V35 Difficult to relax	0.77	0.82	0.17	0.09	−0.18
V19 Worried	0.64	0.72	−0.14	0.27	−0.07
V31 Avoid smiling	0.65	0.69	−0.10	0.00	0.26
V46 Unable to enjoy people’s company	0.63	0.66	0.35	−0.20	−0.05
V47 Life unsatisfying	0.79	0.63	0.34	−0.08	0.12
V4 Appearance affected	0.63	0.51	−0.20	0.19	0.39
V45 Financial loss *	0.51	0.38	0.28	0.06	−0.12
Factor 2. *Functional Limitations*					
V49 Unable to work	0.54	0.05	0.75	−0.02	−0.10
V48 Unable to function	0.42	−0.18	0.69	−0.06	0.15
V43 Difficulty doing job	0.67	0.17	0.62	0.20	−0.16
V40 Less tolerant of others	0.49	−0.08	0.58	0.00	0.19
V39 Avoiding going out	0.62	0.14	0.58	−0.14	0.28
V37 Concentration affected	0.73	0.31	0.56	0.20	−0.20
V42 Irritable with others	0.68	0.16	0.55	0.18	−0.01
V32 Interrupt meals	0.72	−0.11	0.52	0.41	0.19
V3 Noticed tooth doesn’t look right	0.30	0.34	−0.50	0.49	0.07
V29 Diet unsatisfactory *	0.54	−0.14	0.46	0.46	−0.05
V28 Avoid eating *	0.60	−0.08	0.43	0.30	0.20
V33 Sleep interrupted *	0.56	−0.11	0.41	0.34	0.15
V41 Trouble getting on with others *	0.64	0.40	0.41	−0.21	0.28
V11 Sore jaw *	0.44	−0.09	0.37	0.20	0.15
V7 Food catching *	0.30	−0.01	0.30	0.15	−0.05
Factor 3. *Orofacial Pain*					
V13 Sensitive teeth	0.41	−0.12	−0.03	0.78	−0.08
V14 Toothache	0.43	−0.21	0.11	0.70	0.00
V17 Sore spots	0.52	−0.17	0.11	0.69	0.12
V12 Headaches	0.51	0.26	−0.01	0.61	−0.26
V15 Painful gums	0.59	0.06	0.19	0.61	−0.10
V10 Painful aching	0.37	−0.11	0.18	0.58	−0.17
V44 Health worsened	0.72	0.12	0.37	0.57	−0.14
V16 Uncomfortable to eat	0.65	0.13	0.08	0.57	0.12
V1 Difficulty chewing *	0.51	0.11	−0.06	0.48	0.20
V5 Breath stale *	0.29	0.10	−0.35	0.45	0.25
V8 Digestion worse *	0.63	−0.02	0.20	0.41	0.34
V26 Less flavour in food *	0.60	0.11	0.23	0.35	0.14
V6 Taste worse *	0.59	0.09	0.23	0.29	0.23
Factor 4. *Speech Limitations*					
V2 Trouble pronouncing words	0.35	−0.06	0.03	−0.09	0.78
V24 Speech unclear	0.69	0.07	0.44	−0.05	0.57
V25 Others misunderstood	0.64	0.03	0.49	−0.12	0.57
V27 Unable to brush teeth	0.41	0.00	0.10	0.06	0.51
Total variance explained (%)	34.7	34.7	10.5	5.1	4.1

Notes: Extraction method: Principal Component Analysis; Rotation method in the four-factor solution: Promax with Kaiser Normalization; * Item did not have any salient (≥0.50) loading in the four-factor solution; For each item, maximal loading in the four-factor solution is shaded; OHIP-LT: Oral Health Impact Profile, Lithuanian version.

**Table 3:** Results of confirmatory factor analysis: standardized factor loadings in the single-factor (37 items) and four-factor (34 items) solution of the OHIP-LT.

Factor Name and Item	Standardized Factor Loadings				
	Single-Factor Solution	Four-Factor Solution			
		Factor 1	Factor 2	Factor 3	Factor 4
Factor 1. *Psychosocial Impact*					
V34 Upset	0.78	0.83			
V22 Uncomfortable about appearance	0.76	0.90			
V20 Self-conscious	0.77	0.88			
V21 Miserable	0.83	0.91			
V36 Depressed	0.80	0.82			
V38 Been embarrassed	0.77	0.80			
V23 Tense	0.76	0.81			
V35 Difficult to relax	0.84	0.81			
V19 Worried	0.69	0.73			
V31 Avoid smiling	0.71	0.73			
V46 Unable to enjoy people’s company	0.68	0.67			
V47 Life unsatisfying	0.82	0.50	0.48		
V4 Appearance affected	0.63	0.62			
V45 Financial loss *	0.50				
Factor 2. *Functional Limitations*					
V49 Unable to work	0.47		0.67		
V48 Unable to function ^#^			0.49		
V43 Difficulty doing job	0.59		0.75		
V40 Less tolerant of others ^#^			0.54		
V39 Avoiding going out	0.55		0.70		
V37 Concentration affected	0.68	0.27	0.57		
V42 Irritable with others	0.60		0.80		
V32 Interrupt meals	0.57		0.43	0.44	
V3 Noticed tooth doesn’t look right ^#^			x		
V29 Diet unsatisfactory *	0.40				
V28 Avoid eating *	0.48				
V33 Sleep interrupted *	0.44				
V41 Trouble getting on with others *	0.63				
V11 Sore jaw ^#^ *					
V7 Food catching ^#^ *					
Factor 3. *Orofacial Pain*					
V13 Sensitive teeth ^#^				0.61	
V14 Toothache ^#^				0.65	
V17 Sore spots	0.38			0.69	
V12 Headaches	0.47			0.55	
V15 Painful gums	0.48			0.71	
V10 Painful aching ^#^				0.57	
V44 Health worsened	0.61		0.29	0.56	
V16 Uncomfortable to eat	0.56			0.65	
V1 Difficulty chewing *	0.44				
V5 Breath stale ^#^ *					
V8 Digestion worse *	0.51				
V26 Less flavour in food *	0.51				
Factor 4. *Speech Limitations*					
V2 Trouble pronouncing words ^#^					0.51
V24 Speech unclear	0.60				0.98
V25 Others misunderstood	0.54		0.17		0.70
V27 Unable to brush teeth ^#^					0.46

Notes: Estimation method: Maximum Likelihood; ^#^ Items with loading <0.50 in the EFA single-factor solution; * Items with all loadings <0.50 in the EFA four-factor solution; OHIP-LT: Oral Health Impact Profile, Lithuanian version.

**Table 4:** Cronbach’s alphas, and intraclass correlation coefficients in the total OHIP-LT and in its subscales defined by the four-factor solution.

Scale/Subscale	Number of Items	Cronbach’s Alpha	Intraclass Correlation Coefficient	
			Value	(95% CI)
Total scale	46 34 *	0.96 0.95	0.95 0.90	(0.93; 0.96) (0.87; 0.93)
Factor 1. *Psychosocial Impact*	14 13 *	0.95 0.96	0.94 0.94	(0.93; 0.97) (0.91; 0.96)
Factor 2. *Functional Limitations*	15 9 *	0.88 0.84	0.87 0.54	(0.83; 0.91) (0.35; 0.69)
Factor 3. *Orofacial Pain*	13 8 *	0.87 0.85	0.81 0.78	(0.73; 0.87) (0.69; 0.85)
Factor 4. *Speech Limitations*	4	0.75	0.48	(0.25; 0.66)

Notes: OHIP-LT: Oral Health Impact Profile, Lithuanian version; * Estimations for the scale and subscales of items that have all salient (≥0.50) loadings.

**Table 5:** Results of convergent validity analysis: correlation between the estimates of OHIP-LT and self-reported dental health scores.

Estimate	Spearman’s ρ and Significance
Percentage of answers “fairly often” or “very often”	0.74 (*p* < 0.001)
Sum score	0.71 (*p* < 0.001)
General Factor ^a^	0.70 (*p* < 0.001)
Factor 1. *Psychosocial Impacts*	0.74 (*p* < 0.001)
Factor 2. *Functional Limitations*	0.29 (*p* = 0.019)
Factor 3. *Orofacial Pain*	0.51 (*p* < 0.001)
Factor 4. *Speech Limitations*	0.23 (*p* = 0.059)

Notes: OHIP-LT: Oral Health Impact Profile, Lithuanian version; ^a^ A factor from the single-factor solution.

**Table 6:** Results of discriminative validity analysis: means ± SE of the OHIP-LT estimates, by demographic and clinical groups of subjects.

Group of Subjects	*n*	Percentage of Response Options “Fairly Often” and “Very Often”	Sum Score	General Factor ^a^	Factor 1 *Psychosocial Impacts*	Factor 2 *Functional Limitations*	Factor 3 *Orofacial Pain*	Factor 4 *Speech Limitations*
All subjects	67	23.17 ± 2.21	35.23 ± 1.96	0.00 ± 0.12	0.00 ± 0.12	0.00 ± 0.12	0.00 ± 0.12	0.00 ± 0.12
Sex:								
male	21	20.91 ± 3.97	32.32 ± 3.47	−0.19 ± 0.22	−0.36 ± 0.20	−0.14 ± 0.22	0.14 ± 0.19	−0.0 ± 0.21
female	46	24.20 ± 2.67	36.55 ± 2.37	0.09 ± 0.15	0.17 ± 0.15	0.06 ± 0.15	−0.06 ± 0.15	0.01 ± 0.15
		*p* = 0.405	*p* = 0.327	*p* = 0.262	*p* = **0.048**	*p* = 0.311	*p* = 0.311	*p* = 0.882
Age:								
≤40 yrs	27	27.86 ± 3.20	39.73 ± 2.81	0.26 ± 0.18	0.44 ± 0.18	0.07 ± 0.19	−0.01 ± 0.20	0.15 ± 0.21
>40 yrs	40	20.00 ± 2.92	32.19 ± 2.60	−0.18 ± 0.16	−0.30 ± 0.15	−0.04 ± 0.16	0.01 ± 0.15	−0.10 ± 0.15
		*p* = **0.020**	*p* = **0.034**	*p* = **0.040**	*p* = **0.004**	*p* = 0.458	*p* = 0.949	*p* = 0.255
Presence of CAL ≥ 5 mm:								
≤30%	52	18.60 ± 1.85	31.86 ± 1.86	−0.21 ± 0.11	−0.13 ± 0.13	−0.12 ± 0.11	−0.23 ± 0.12	−0.17 ± 0.12
>30%	15	38.99 ± 6.01	46.92 ± 4.95	0.71 ± 0.32	0.46 ± 0.26	0.41 ± 0.37	0.82 ± 0.28	0.59 ± 0.30
		*p* = **0.003**	*p* = **0.005**	*p* = **0.010**	*p* = **0.037**	*p* = 0.052	*p* = **0.001**	*p* = **0.020**
Spacing:								
no spacing	36	16.91 ± 2.30	30.33 ± 2.13	−0.31 ± 0.13	−0.28 ± 0.15	−0.25 ± 0.11	−0.15 ± 0.15	−0.19 ± 0.14
had spacing	31	30.43 ± 3.56	40.92 ± 3.17	0.36 ± 0.20	0.33 ± 0.18	0.29 ± 0.22	0.17 ± 0.19	0.22 ± 0.20
		*p* = **0.004**	*p* = **0.010**	*p* = **0.013**	*p* = **0.009**	*p* = 0.105	*p* = 0.213	*p* = 0.155

Notes: OHIP-LT: Oral Health Impact Profile, Lithuanian version; SE: standard error; CAL: Clinical attachment loss; ^a^ A factor from the single-factor solution; Values *p* < 0.05 are in bold (Mann-Whitney U test).

## Data Availability

The dataset supporting the conclusions of this article is available from the corresponding author on reasonable request.

## Abbreviations

CAL: Clinical attachment loss; CFA: Confirmatory factor analysis; CFI: Comparative fit index; CI: Confidence limits; EFA: Exploratory factor analysis; ICC: Intraclass correlation coefficient; OHIP: Oral Health Impact Profile; OHIP-Lt: Lithuanian version of the OHIP; OHRQoL: Oral Health Related Quality of Life; RMSEA: Root mean square error of approximation; TLI: Tucker-Lewis index.
